# Serrated polyposis syndrome: defining the epidemiology and predicting the risk of dysplasia

**DOI:** 10.1186/s12876-024-03247-2

**Published:** 2024-05-16

**Authors:** Natalie R. Dierick, Brian D. Nicholson, Thomas R. Fanshawe, Praka Sundaralingam, Stuart N. Kostalas

**Affiliations:** 1 School of Clinical Medicine, University of New South Wales, Rural Clinical Campus, Port Macquarie, Kensington, NSW Australia; 2 Port Macquarie Gastroenterology, Port Macquarie, NSW Australia; 3 Nuffield Department of Primary Care Health Sciences, University of Oxford, Oxford, UK

**Keywords:** Serrated polyposis syndrome, Epidemiology, Colorectal cancer

## Abstract

**Background:**

Serrated polyposis syndrome is the most common polyposis syndrome that has neoplastic potential. However, the natural history, genetic basis, and risk of dysplasia and neoplasia of serrated polyposis syndrome are incompletely understood. The objective of this study is to define the epidemiology of serrated polyposis syndrome. Using this data, we aim to evaluate candidate variables for predicting the risk of dysplasia and neoplasia in sessile serrated lesions found in serrated polyposis syndrome patients. Finally, we aim to use this data to create and evaluate clinical prediction models for accuracy in predicting dysplastic sessile serrated lesions in serrated polyposis syndrome patients.

**Methods:**

This was a regional Australian single-centre retrospective cohort study. Data was prospectively collected data from the clinical record database of a regional Australian gastroenterology practice. All patients undergoing colonoscopy at Port Macquarie Gastroenterology between January 2015 and September 2021 were screened for this study. Collected data included patient demographic, endoscopic, and histopathological findings. Clinical and endoscopic multivariate logistic regression models were created to predict dysplastic sessile serrated lesions. Model performance was examined using the area under the receiver operating curve.

**Results:**

In total 8401 patients underwent a colonoscopy procedure during the study period. Serrated polyposis syndrome was diagnosed in 247, representing a prevalence of 2.94% (mean age 67.15 years, 62.75% female). Logistic regression identified; older age at serrated polyposis syndrome diagnosis, a personal history of colorectal cancer, size of the largest sessile serrated lesions removed, and total sessile serrated lesions count as predictors of dysplastic sessile serrated lesions. The clinical and endoscopic model had an area under the receiver operating curve of 0.75.

**Conclusion:**

Serrated polyposis syndrome is more common than previously described. The clinical and endoscopic variables identified in logistic regression have acceptable accuracy in predicting the risk of dysplasia, however other populations need to be studied to achieve generalisability and improve model performance.

**Supplementary Information:**

The online version contains supplementary material available at 10.1186/s12876-024-03247-2.

## Background

Serrated polyposis syndrome (SPS) is characterised by multiple serrated lesions throughout the colorectum, and carries 19.9% risk of colorectal cancer (CRC) development [1]. Prevention of CRC involves timely detection and removal of serrated lesions, however little understood about the aetiology and predictors of SPS.

SPS is the most common colonic polyposis syndrome, with an estimated prevalence of 1 in 111 in screening populations [2]. SPS is characterised by multiple colorectal serrated lesions, which include hyperplastic polyps (HPs), sessile serrated lesions (SSLs), and traditional serrated adenomas (TSAs). These lesions arise throughout the colorectum via the serrated neoplasia pathway. SPS likely represents a disease spectrum with a high degree of heterogeneity, influenced by genetic predisposition and environmental factors. As no definite genetic aetiology for SPS has been identified, clinicians must solely rely upon the World Health Organisation’s (WHO) clinical criteria, defined by accumulative presence of:

1. ≥ 5 serrated lesions proximal to the rectum, all being ≥ 5 mm in size, with ≥ 2 being ≥ 10 mm in size and/or.
2. > 20 serrated lesions of any size distributed throughout the large bowel, with ≥ 5 being proximal to the rectum [3].

Despite its neoplastic potential, SPS is suspected to be underdiagnosed, due to unavailability of previous pathology, endoscopy reports, and failure of clinicians to correctly apply the diagnostic criteria [4]. Understanding the clinical and endoscopic risk factors of SPS, will assist clinicians to avoid underdiagnosis. However, to date there is a lack of data regarding the prevalence of SPS globally. A significant limitation of most studies assessing clinical factors are the small sample sizes, inconsistent nomenclature, and changing endoscopic practices [5]^,^ [6].

SSLs pose a significant challenge to clinicians for several reasons. Firstly, a disproportionate number of interval CRC is linked to the serrated neoplasia pathway. Secondly, SSLs have been shown to be difficult to detect, due to their inconspicuous colour, flat or sessile morphology, paucity of surface vessels, and camouflaging mucus cap. This has led to variation in detection rates among endoscopists. Histopathologists are also challenged due to microscopic similarities shared with other serrated lesions. Finally, due to their difficult to demarcate and indistinct boarders, SSLs are often incompletely resected [7, 8].

A comprehensive dataset from a large sample size would help to better understand the risk factors for SPS and dysplastic SSLs (dSSLs). Using this data to create a clinical prediction model could assist clinicians in providing personalised treatment and targeted primary prevention [9].

### Research question

1. What are the clinical and endoscopic factors of SPS present in a regional Australian cohort?
2. Using clinical prediction models based on collected candidate variables, can we accurately predict dSSLs diagnosis in SPS patients?

### Research aims

1. To identify patients who meet the WHO 2019 criteria for SPS from a cohort of adult patients (≥ 18 years of age) undergoing colonoscopy (for any indication) in a region Australian setting. From the identified cohort, evaluate and define the epidemiology (clinical and endoscopic factors) of SPS.
2. Using data collected from this cohort, evaluate candidate variables for predicting the risk of dysplasia/neoplasia in SSLs.
3. To create and evaluate clinical prediction models, specifically prognostic prediction models, for accuracy in diagnosing dSSLs in SPS patients.

## Methodology

### Study design

This was a single-centre study with prospectively collected data from the clinical record database of a regional Australian gastroenterology practice. All patients undergoing colonoscopy at Port Macquarie Gastroenterology between January 2015 and September 2021 for any reason were screened for this study. Verbal consent for data collection was obtained from patients at the time of consent for colonoscopy. Patients that met the WHO 2019 criteria for SPS diagnosis during this period were enrolled to participate the study. Routine pre-endoscopy demographic details, along with medical history, examination details, and endoscopic findings were recorded for all patients in the practice software (Genie version 9.2.2., Magic Carpet Software Solutions). This data was recorded in an excel database.

### Target population

*Inclusion criteria*: Eligible patients were those aged 18 years or older undergoing endoscopic investigations (colonoscopy) for any reason.

*Exclusion criteria*: Patients who refused to consent to data collection.

### Definitions

In all cases the diagnosis of SPS was made in accordance with the WHO 2019 criteria. In patients who had undergone a colonoscopy prior to the study period, previously resected serrated lesions were used to qualify for SPS diagnosis, provided a histopathological report confirmed the presence, size, and location of the serrated lesions. Some participants during the study period met the inclusion criteria after a surveillance colonoscopy as SPS is an accumulative diagnosis. No subject data is included twice. For patients diagnosed with SPS during the study period undergoing surveillance have a recorded accumulative SSL count along with the number of surveillance colonoscopies they have had in the dataset.

### Endoscopy

Colonoscopies were conducted in accordance with recent guidelines on quality indicators in colonoscopy [10]. Patients included in the study were given split polyethylene glycol (PEG) bowel preparation (MoviPrep® - Norgine pharmaceuticals) or sodium picosulphate if intolerant.

### Histopathology processing

Histopathology was processed and stained using standard methods and were polyps evaluated according to the Vienna Classification [11] and classified according to the WHO SPS subtype definitions [12]. Neoplasia was defined and classified according to the current WHO classification [13]. dSSLs were reviewed by specialist gastrointestinal histopathologists.

### Predictor variables; selection, measurement, and timing

A review of the established literature on SPS and dSSLs enabled identification of potential clinical and endoscopic predictor variables. These included age, sex, smoking diabetes, ethanol consumption, family history of CRC, personal history of CRC, index colonoscopy, FOBT result, number of SSLs at diagnostic endoscopy, other types of adenomas present, size of largest SSL (mm) number of SSLs > 10 mm, total number of SSLs, average number of SSLs detected per surveillance colonoscopy, and DSSL morphology. Clinical records of patients identified with SPS were reviewed to extract demographic, clinical, endoscopic, and histopathological factors. All the variables and their quantification were pre-specified in the statistical analysis plan (supplementary file 1).

### Sample size

Using an events per variable predictor (EVP) of 10 and total of 17 candidate variables a sample size of 170 events was calculated. Following model development, a Cox-Snell R^2^ value of 0.15 was obtained. This was used to calculate a sample size of 849 events.

### Statistical analysis

Data was analysed using R software. Descriptive statistics were used to outline the clinical epidemiology. The relationship between dysplasia and clinical, endoscopic, or histopathological categorical variables were evaluated using Fisher’s exact test. The relationship between dysplasia and clinical, endoscopic, or histopathological continuous variables were evaluated using a two-sided sample t-test. Modelling strategies included stepwise selection and penalised estimation. These were used to identify clinical and endoscopic predictors of risk of dysplasia or CRC. Model outcomes included area under the receiver operating characteristic (AUROC) data used to highlight model estimation suitability.

### Ethics

Ethics approval for this study includes *Refence Number: EQ C1A 19 052* provided by the Social Sciences and Humanities Inter-divisional Research Ethics Committee at the University of Oxford, and *Reference Number: P344* provided by the QIMR Berghofer – Human Research Ethics Committee.

## Results

### Recruitment and study population

Between January 2015 and September 2021, 8401 patients who had colonoscopies performed at Port Macquarie Gastroenterology were screened for study inclusion. Patients under the age of 18 years [6] or declining consent (0) were excluded from the study. Of these study participants, 247 patients (2.94%) were identified who met the 2019 WHO diagnostic criteria for SPS and were included in the final analysis.

### Clinical characteristics

Clinical characteristics of patients diagnosed with SPS are outlined in Table 1. Majority of patients were female (62.75%). All the patients fell into category 1 of WHO 2019 SPS clinical criterion, with 3.64% of SPS patients also satisfying category 2. SPS patients were diagnosed at a mean age of 67.15 years (SD 13.31 years). Of the patients, 29.96% had 1st degree family history of CRC and 10.53% had 2nd degree family history of CRC. Only 9.72% of patients had a history of CRC, with 5.67% prior to the diagnosis, 3.24% at the time of diagnosis, and 0.81% after the diagnosis. Majority of patients had previous colonoscopic investigations, with only 19.84% of patients diagnosed from an index colonoscopy. Majority of patients did not perform an faecal occult blood test (FOBT) (83.00%), with only 12.55% of patients having a positive FOBT within 3 months of their diagnostic colonoscopy. Of the patients, 4.86% were current smokers, 23.08% ex-smokers, and 72.06% lifelong non-smokers. Of the patients, 6.48% of patients were diabetic and 18.22% of the SPS patients consumed > 30gm of ethanol per day.

**Table 1:** Clinical characteristics of SPS patients

Clinical characteristics	Total SPS cohort (247)
**Sex** (count & %)	
Male	92 (37.24%)
Female	155 (62.75%)
**WHO diagnostic category**	
≥ 5 with 2 ≥ 10mm	247 (100%)
≥ 20 serrated lesions	9 (3.64%)
**Age at SPS diagnosis** (years)	
Mean	67.15
SD	13.31
Range	23–91
**Family History of CRC** (count & %)	
1st degree	74 (29.96%)
2nd degree	26 (10.53%)
No	134 (54.25%)
Missing data	13 (5.26%)
**Previous History of CRC** (count & %)	
CRC prior to Dx of SPS	14 (5.67%)
CRC at time of SPS Dx	8 (3.24%)
CRC post SPS Dx	2 (0.81%)
No CRC	223 (90.28%)
**Index colonoscopy** (count & %)	
Yes	49 (19.84%)
No	197 (79.76%)
Unclear	1 (0.40%)
**FOBT status** (count & %)	
Positive	31 (12.55%)
Negative within 3 months	11 (4.45%)
Not Performed	205 (83.00%)
**Smoking status** (count & %)	
Yes	12 (4.86%)
No	178 (72.06%)
Ex-Smoker	57 (23.08%)
**Diabetes** (count & %)	
Yes	16 (6.48%)
No	231 (93.52%)
**Ethanol intake** (> 30gm per day) (count & %)	
Yes	45 (18.22%)
No	202 (81.78%)

### Endoscopic characteristics

Endoscopic characteristics of patients diagnosed with SPS are outlined in Table 2. Dysplasia was found in 27.94% of SSLs and cancer was identified in 5.67% of SSLs. Of the patients, the mean size of the largest SSL at diagnosis was 18.05 mm (SD 7.51 mm). The mean number of SSLs found endoscopically at diagnosis was 8.17 (SD 4.09). The mean number of histopathologically confirmed SSLs at diagnosis was 5.83 (SD 2.85). The mean number of SSLs > 10 mm was 4.73 (SD 2.18). The majority of patients had other types of adenomas (79.35%), with tubular adenomas being the most common (52.63%). The mean total number of histopathologically diagnosed SSLs in the cohort was 9.17 (SD 7.23), with a mean average number of 1.80 SSLs detected per surveillance colonoscopy (SD 2.06).

**Table 2:** Endoscopic and histopathological characteristics of SPS patients

Endoscopic/histopathological characteristics	Total SPS cohort (247)
**Dysplasia** (count & %)	
Yes	68 (27.53%)
No	179 (72.47%)
**Neoplasia in SSL** (count & %)	
Yes	14 (5.67%)
No	233 (94.33%)
**Size of largest SSL at Diagnosis** (mm)	
Mean	18.05
SD	7.51
Range	6–75
**Number of SSLs at Diagnosis endoscopy**	
Mean	8.17
SD	4.09
Range	3–30
**Number of SSLs histologically confirmed at Diagnosis**	
Mean	5.83
SD	2.85
Range	1–23
**Number of SSLs > 10mm at Diagnosis**	
Mean	4.73
SD	2.18
Range	0–15
**Other types of adenomas** (count & %)	
Yes	196 (79.35%)
No	51 (20.65%)
**Breakdown of other types of polyps in SPS patients** (count & %)	
TA	130 (52.63%)
TVA/VA	17 (6.88%)
HP	4 (1.62%)
Mixed	44 (17.81%)
Other	2 (0.81%)
NA	50 (20.24%)
**Total number of SSLs histopathologically confirmed**	
Mean	9.17
SD	7.23
Range	1–68
**Average number of SSLs detected per surveillance colonoscopy**	
Mean	1.80
SD	2.06
Range	0–11

### dSSL characteristics

Table 3 outlines the endoscopic and histopathological characteristics of dSSLs. Most of the dSSLs were located in the right colon (88.24%), in keeping with the well described colonic location for SSLs [14]. All resected dysplastic lesions were of Paris IIa morphology, which is consistent with the well-defined endoscopic appearance of SSLs [15]. Paris 0-Is component was found in 30.88% of SSLs. Neoplasia was found in 20.59% of dSSLs.

**Table 3:** Lesion characteristics of dysplastic SSLs in SPS patients

Endoscopic/Histopathological Characteristics	Total SPS cohort (68)
**Size of DSSL** (mm)	
Mean	15.95
SD	10.22
Range	5–75
**Colonic Location** (count & %)	
Right Colon	60 (88.24%)
Left Colon	8 (11.76%)
**Paris morphology** (count & %)	
IIa	65 (95.59%)
Missing data	3 (4.41%)
**Paris 0-Is component** (count & %)	
Yes	21 (30.88%)
No	40 (58.82%)
Missing data	7 (10.29%)
**Neoplastic component** (count & %)	
Yes	14 (20.59%)
No	54 (79.41%)

### Clinical, endoscopic, and histopathological characteristics of SPS patients with and without Dysplasia

**Fig. 1:**
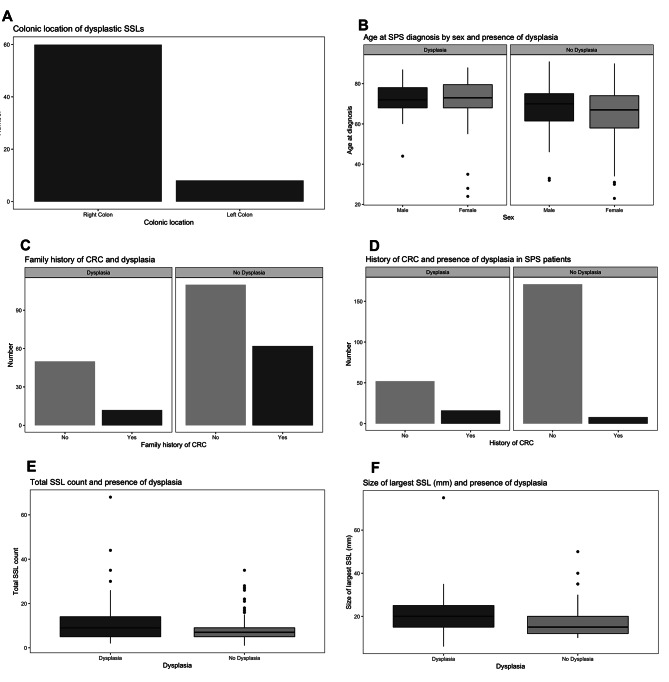
Bar Charts and a Forest Plots demonstrating the relationship between dysplastic SSLs and studied characteristic. **A** Colonic location of DSSLs. **B** Age at SPS diagnosis subdivided by sex and presence of dysplasia. **C** Family history of CRC subdivided by presence of dysplasia. **D** Patient history of CRC subdivided by presence of dysplasia. **E** Total SSL count and presence of dysplasia. **F** Size of largest SSL (mm) and presence of dysplasia

The relationship between dSSLs and the clinical, endoscopic, and histopathological characteristics of SPS are outlined in Fig. 1. There was an association between dysplasia and age, with SPS patient > 55-years-old more frequently had dysplasia (OR 0.32, 95% CI 0.09–0.86, *p* = 0.02039). Having a 1st degree relative with CRC was found to be a protective factor against dysplasia (OR 0.43, 95% CI 0.19–0.89, *p* = 0.0169). Dysplasia was more frequent among patients with a personal history of CRC (OR 6.51, 95% CI 2.47–18.64, *p* = 0.0001). Dysplasia was associated with increasing SSL number, with dysplasia more frequently observed in patients with a lifetime count > 20 SSLs (OR 3.24, 95% CI 1.06–10.15, *p* = 0.0228). In addition, an increase in the average number of SSLs detected during surveillance colonoscopies was associated with an increasing risk of dysplasia (*p* = 0.0248, 95% CI 0.10–1.50). Increasing SSL size at diagnosis was associated with an increased risk of dysplasia (*p* = 0.0090, 95% CI 0.08–5.76).

### Prediction models of dysplasia

A logistic model was created from the SPS dataset to predict the risk of dysplasia (Table 4). Logistic regression analysis was performed from clinical and endoscopic variables selected from stepwise calculations. Patients with unknown family history of CRC and previous personal history of CRC were removed from the final analysis, leaving 233 observations. In the model the variables that were statistically significant in predicting an increased risk of dysplasia were age at diagnosis (OR 0.97, 95% CI 0.95–1.00, *p* = 0.036), a 1st degree relative with CRC (OR 2.16, 95% CI 1.01–4.93, *p* = 0.046), a history of CRC (OR 0.16, 95% CI 0.05–0.49, *p* = 0.001), the size of the largest SSL removed at diagnosis (OR 0.95, 95% CI 0.90–0.99, *p* = 0.017), and lifetime total SSL count (OR 0.94, 95% CI 0.90–0.98, *p* = 0.006).

**Table 4:** Relationship between dysplasia and clinical and endoscopic variables controlling for other variables

Clinical and endoscopic variables	Odds Ratio	95% CI	Chi2	*P*-value
**Female sex**	0.62	0.30–1.25	230.51	0.188
**Age at diagnosis**	0.97	0.94–1.00	230.51	0.022*
**Family history of CRC**	2.01	0.97–4.41	230.51	0.060
**History of CRC**	0.16	0.05–0.45	230.51	< 0.001*
**Size of largest SSL**	0.96	0.92–1.00	230.51	0.056
**Total SSL count**	0.94	0.90–0.98	230.51	0.006*

*Significant Value

### Model analysis

The final clinical and endoscopic model were used to create receiver operating characteristic (ROC) curves. The model had an area under ROC (AUROC) of 0.75. The clinical and endoscopic model yielded a sensitivity of 32.26% and specificity of 93.60% (corresponding to a threshold of 0.5).

## Discussion

In this single centre prospective cohort study, we assessed the clinical, endoscopic, and histological characteristics of SPS and risk factors for dSSLs in SPS patients. Statistical analysis demonstrated an increased risk of dysplasia with increasing SSL size at diagnostic endoscopy, previous history of CRC, total lifetime SSL count, and increasing age at diagnosis, when controlling for confounding factors.

### Epidemiological findings

The prevalence of SPS is not well understood and is thought to be much higher than previously reported in the literature. In CRC screening cohorts the prevalence of SPS ranged between 0 and 0.8% [2, 16–18] and has previously been reported in an Australian cohort study to be 0.26% [19]. This cohort study included patients undergoing colonoscopy for any reason and found the prevalence of SPS to be 2.94%. In addition, our cohort had a higher rate of dysplasia (27.53%) in comparison to the existing literature (19.5%). This finding is hypothesised to be caused by high levels of detection bias due to significant misdiagnosis rates in previous studies. This is supported by our finding that majority patients had undergone previous colonoscopies prior to their SPS diagnosis. In addition, over the last decade awareness of SPS has improved among endoscopists, international quality standards for colonoscopy performance have been established, endoscopic image technology has advanced, systematic tracking of serrated lesions have been implemented, split bowel preparation has been adopted, and there has been widespread adoption of population-based screening programs [20, 21]. This has enabled detection of previously “missed” lesions. However, this increased SPS prevalence rate could also be due to a true increase in population prevalence, or a unique patient population. Sampling bias and confounding factors may have affected the results as study participants were recruited from a single centre in a regional Australian town. A multicentre study incorporating different demographics would enable a more robust and reliable analysis.

Consistent with the literature regarding patients with SPS, our results confirm that this syndrome occurs between 50 and 75 years in both males and females [2]. Most patients in this study developed SPS developed over the age of 50 years and did not have a family history of CRC. This finding suggests that SPS is not an inherited syndrome. SPS appears to be a complex disorder with a poorly defined etiological pathway.

### Clinicopathological predictors of dysplasia in SPS patients

Over the last decade our knowledge regarding CRC risk in SPS patients has significantly improved. However, the clinical and endoscopic risk factors of dysplasia development remain poorly understood.

Interestingly, family history of CRC in a first degree relative was associated with a statistically significant decrease in the risk of dSSLs in SPS patients. This may represent a confounding effect, as patients with a family history of CRC may have undergone colonoscopy screening at a younger age, enabling the early detection of lesions before dysplasia has the chance to develop.

Increasing SSL size removed at diagnostic endoscopy was found to be a statistically significant factor in dysplasia development. This association may be explained by the theory that SSLs are relatively slow growing, but once dysplasia is identified, progression to malignancy is rapid. Cross-sectional studies indicate a 15-year interval between SSLs without cytological dysplasia to CRC and a medial 10-year interval between SSLs with low-grade cytological dysplasia and CRC. However, Bettington et al., found no significant age difference of patients with dSSLs verses those with CRC [14], and Limketkai et al., found that CRC developed 8 to 24 months after incomplete SSL resection. The underlying mechanism of this rapid progression from dysplasia to malignancy is thought be microsatellite instability (MSI) [22].

A total lifetime count of SSLs was identified as statistically significant factor that increased dSSLs risk. This may suggest an underlying genetic predisposition to disordered growth, however SPS does not have typical features of dominant inheritance [23]. Further studies assess environmental factors are needed.

### Modelling outcomes of SPS patients with dSSLs and application to clinical practice

The objective of the SPS prognostic prediction model is to accurately predict dSSLs in SPS patients. To determine model performance, we performed predictions on a hypothetical group of 1000 SPS patients based on SPS population prevalence of dysplasia and model sensitivity and specificity (Table 5).

**Table 5:** Worked example of clinical and endoscopic factors model

		Patients with DSSLs			
		Histology positive	Histology negative	Prevalence = 27.53%	Accuracy = 76.70%
**Model Prediction**	Predicts dysplasia	True positive = 89	False positive = 46	Positive predictive value = 65.93%	False discovery rate = 34.07%
	Predicts no dysplasia	False negative = 187	True negative = 678	False omission rate = 21.62%	Negative predictive value = 78.38%
		Sensitivity = 32.25%	False positive rate = 6.35%		
		False negative rate = 67.75%	Specificity = 93.60%		

If a guideline to personalise surveillance intervals was introduced based on this prediction model, then 187 high-risk patients would be incorrectly changed to longer surveillance intervals. Furthermore, 46 low risk patients would be surveyed annually. However, because this is the current standard of care it is a low risk of harm. The correct surveillance interval would be set for 767 patients. Given the low risk of more frequent surveillance, that would leave 18.7% of SPS patients at high risk of a poor outcome from this change in surveillance interval. Conversely, 67.8% of patient would be able to have their colonoscopy surveillance burden reduced by 66%. Although the clinical and endoscopic variables identified in logistic regression have acceptable accuracy (76.70%) in predicting the risk of dysplasia, model performance is insufficient to recommend use in clinical practice. Further studies involving multicentre cohorts and a larger sample size will improve modelling performance and achieve generalisability.

### Strengths

In comparison to previous studies, we believe our research has several strengths. Firstly, our study represents a large single centre cohort of SPS patients based on the current WHO diagnostic criteria. Secondly, detailed endoscopic and histopathological information was collected from highly experienced gastroenterologists and analysed by specialised gastrointestinal histopathologists.

### Limitations

The majority of colonoscopies in this study are performed by a single gastroenterologist, who has a high SSL detection rate (47%). This may have introduced a detection bias. Applying this study design to multicentre cohort, would enable a better understanding of the epidemiology. Generalisability of the study is limited as resected serrated lesions prior to the defined study time were used to qualify for an SPS diagnosis. In addition, the reproducibility of the study is compromised as the screened cohort includes a mix of various indications for colonoscopy. This decision was made to reflect real world colonoscopy practice.

### Future directions

SPS patients should have colonoscopies performed by an endoscopist with a high SSL detection rate. This will prevent missed lesions and decrease the risk of dysplasia and interval CRC in SPS patients.

Current international surveillance colonoscopy guidelines are homogenous, recommending that all SPS patients undergo surveillance every 1–3 years [24]. These guidelines aim to prevent interval colorectal cancer, however without risk stratification all SPS patients incur a substantial colonoscopy burden. Furthermore, the increasing rate of SPS diagnosis, necessitates increased and more frequent surveillance colonoscopies, which places significant strain on the healthcare system resources. Given the slight increased risk of CRC development during surveillance (2.8%), it may be safe to de-intensity surveillance intervals for low risk SPS patients after initial polyp clearance [1]. However, a study by Bleijenberg et al., found that an individual’s polyp burden, nature, and size varied between surveillance colonoscopies, and did not follow an upward or downtrend trend [25]. This suggests that SPS patients are at increased risk of developing dysplastic and neoplastic SSLs long after their diagnosis and after clearance of their initial polyp burden. Our study and several recent studies suggest that dysplasia and CRC risk are dependent on patient-specific risk factors^13,18,19^. Personalised surveillance intervals could be implemented by utilising individualised risk stratification and clinical prediction models. This may allow clinicians to better allocate healthcare resources to high-risk SPS patients to prevent interval CRC and prevent overtreatment, reducing the colonoscopy burden for low-risk patients [26].

## Conclusion

This study demonstrated that the rate of SPS is much higher (2.94%) than previously reported (< 1%). This information should alert clinicians to the frequency and neoplastic potential of SPS and encourage endoscopist to thoughtfully look for SSLs and SPS.

This study has enabled a better understanding of the risk factors of SPS and dSSLs. Clinical prediction models provided acceptable prognostic prediction for the risk of dysplasia in patients with SPS. However, this study methodology needs to be applied to other populations to achieve generalisability.

### Electronic supplementary material

Below is the link to the electronic supplementary material.

Supplementary Material 1

Supplementary Material 2

Supplementary Material 3

## Data Availability

The data that support the findings of this study are available from Port Macquarie Gastroenterology, but restrictions apply to the availability of this data, which were used under license for the current study, and so are not publicly available. The data however is available upon reasonable request and with permission. Please contact Stuart Kostalas to access the data. A statistical analysis plan is available (additional file 1).

## Abbreviations

AUROC: Area under the receiver operating characteristic
CI: Confidence interval
CRC: Colorectal cancer
dSSLs: Dysplastic sessile serrated lesions
EVP: Events per variable predictor
FOBT: Faecal occult blood test
HPs: Hyperplasic polyps
KRAS: Kristen rat sarcoma gene
MSI: Microsatellite instability
OR: Odds ratio
PEG: Polyethylene glycol
ROC: Receiver operating characteristic
SD: Standard deviation
SPS: Serrated polyposis syndrome
SSLs: Sessile serrated lesions
TSAs: Traditional serrated adenomas
WHO: World Health Organisation

## References

[1] Muller C, Yamada A, Ikegami S, Haider H, Komaki Y, Komaki F (2022). Risk of Colorectal Cancer in Serrated Polyposis Syndrome: a systematic review and Meta-analysis. Clin Gastroenterol Hepatol.

[2] IJspeert JEG, Bevan R, Senore C, Kaminski MF, Kuipers EJ, Mroz A (2017). Detection rate of serrated polyps and serrated polyposis syndrome in colorectal cancer screening cohorts: a European overview. Gut.

[3] Rosty C, Brosens LA, Dekker E, et al. Serrated polyposis. WHO classification of tumours of the digestive system. 5th ed. World Health Organization; 2019. pp. 532–4.

[4] Van Herwaarden YJ, Pape S, Vink-Börger E, Dura P, Nagengast FM, Epping LSM (2019). Reasons why the diagnosis of serrated polyposis syndrome is missed. Eur J Gastroenterol Hepatol.

[5] Crockett SD, Nagtegaal ID, Terminology. Molecular Features, Epidemiology, and Management of Serrated Colorectal Neoplasia. Gastroenterology [Internet]. 2019;157(4):949–966.e4. 10.1053/j.gastro.2019.06.041. 10.1053/j.gastro.2019.06.041 31323292

[6] IJspeert JEG, Bastiaansen BAJ, Van Leerdam ME, Meijer GA, Van Eeden S, Sanduleanu S (2016). Development and validation of the WASP classification system for optical diagnosis of adenomas, hyperplastic polyps and sessile serrated adenomas/polyps. Gut.

[7] Ijspeert JEG, Vermeulen L, Meijer GA, Dekker E. Serrated neoplasia-role in colorectal carcinogenesis and clinical implications. Nat Rev Gastroenterol Hepatol [Internet]. 2015;12(7):401–9. 10.1038/nrgastro.2015.73. 10.1038/nrgastro.2015.73 25963511

[8] Burgess NG, Pellise M, Nanda KS, Hourigan LF, Zanati SA, Brown GJ (2016). Clinical and endoscopic predictors of cytological dysplasia or cancer in a prospective multicentre study of large sessile serrated adenomas/polyps. Gut.

[9] Davenport JR, Su T, Zhao Z, Coleman HG, Smalley WE, Ness RM (2018). Modifiable lifestyle factors associated with risk of sessile serrated polyps, conventional adenomas and hyperplastic polyps. Gut.

[10] Rex DK, Schoenfeld PS, Cohen J, Pike IM, Adler DG, Fennerty MB (2015). Quality indicators for colonoscopy. Am J Gastroenterol.

[11] Schlemper RJ, Riddell RH, Kato Y, Borchard F, Cooper HS, Dawsey SM et al. The Vienna classification of gastrointestinal epithelial neoplasia. Gut [Internet]. 2000;47(2):251 LP – 255. http://gut.bmj.com/content/47/2/251.abstract. 10.1136/gut.47.2.251 PMC1728018 10896917

[12] Snover D, Ahnen DJ, Burt RW, Bosman FT, Carneiro F, Hruban RH, Theise ND (2010). Serrated polyps of the colon and rectum and serrated (hyperplastic) polyposis. WHO classification of tumours of the digestive system.

[13] Ahadi M, Sokolova A, Brown I, Chou A, Gill AJ (2021). The 2019 World Health Organization classification of appendiceal, colorectal and anal canal tumours: an update and critical assessment. Pathology.

[14] Bettington M, Walker N, Rosty C, Brown I, Clouston A, McKeone D (2017). Clinicopathological and molecular features of sessile serrated adenomas with dysplasia or carcinoma. Gut.

[15] Sweetser S, Smyrk TC, Sinicrope FA (2013). Serrated colon polyps as precursors to colorectal cancer. Clin Gastroenterol Hepatol.

[16] Moreira L, Pellisé M, Carballal S, Bessa X, Ocaña T, Serradesanferm A (2013). High prevalence of serrated polyposis syndrome in fit-based colorectal cancer screening programmes. Gut.

[17] Biswas S, Ellis AJ, Guy R, Savage H, Madronal K, East JE (2013). High prevalence of hyperplastic polyposis syndrome (serrated polyposis) in the NHS bowel cancer screening programme. Gut.

[18] Colussi D, Zagari RM, Morini B, Fabbri M, Montale A, Hassan C (2017). Prevalence of serrated polyposis syndrome in an FIT-based colorectal cancer screening cohort in Italy. Gut.

[19] Wu Y, Mullin A, Stoita A (2017). Clinical predictors for sessile serrated polyposis syndrome: a case control study. World J Gastrointest Endosc.

[20] Mankaney G, Rouphael C, Burke CA. Serrated Polyposis Syndrome. Clin Gastroenterol Hepatol [Internet]. 2020;18(4):777–9. 10.1016/j.cgh.2019.09.006. 10.1016/j.cgh.2019.09.006 31520728

[21] Dekker E, Bleijenberg A, Balaguer F, IJspeert JEG, Bleijenberg AGC, Pellisé M (2020). Update on the World Health Organization Criteria for Diagnosis of Serrated Polyposis Syndrome. Gastroenterology.

[22] Limketkai BN, Lam-Himlin D, Arnold CA, Arnold MA. The cutting edge of serrated polyps: A practical guide to approaching and managing serrated colon polyps. Gastrointest Endosc [Internet]. 2013;77(3):360–75. 10.1016/j.gie.2012.11.013. 10.1016/j.gie.2012.11.013 23410696

[23] Cauley CE, Hassab TH, Feinberg A, Church J (2020). Sessile serrated polyposis: not an inherited syndrome?. Dis Colon Rectum.

[24] Gupta S, Weiss JM, Axell L, Burke CA, Chen L-M, Chung DC et al. NCCN Guidelines Version 2.2023 Genetic/Familial High-Risk Assessment: Colorectal [Internet]. 2nd ed. Darlow S, Dwyer M, editors. 2023. https://www.nccn.org/home/.

[25] Bleijenberg AGC, IJspeert JEG, Hazewinkel Y, Boparai KS, Oppeneer SC, Bastiaansen BAJ et al. The long-term outcomes and natural disease course of serrated polyposis syndrome: over 10 years of prospective follow-up in a specialized center. Gastrointest Endosc [Internet]. 2020;92(5):1098–1107.e1. 10.1016/j.gie.2020.04.068. 10.1016/j.gie.2020.04.068 32360902

[26] Bleijenberg AGC, Ijspeert JEG, Van Herwaarden YJ, Carballal S, Pellisé M, Jung G (2020). Personalised surveillance for serrated polyposis syndrome: results from a prospective 5-year international cohort study. Gut.

