# Supplementary figures and images for: Serrated polyposis syndrome: defining the epidemiology and predicting the risk of dysplasia
Source: BMC Gastroenterol. 2024 May 16;24:167. doi: 10.1186/s12876-024-03247-2 (PMC11100053; doi:10.1186/s12876-024-03247-2)

Figure 1. Flow chart of handling of missing data6


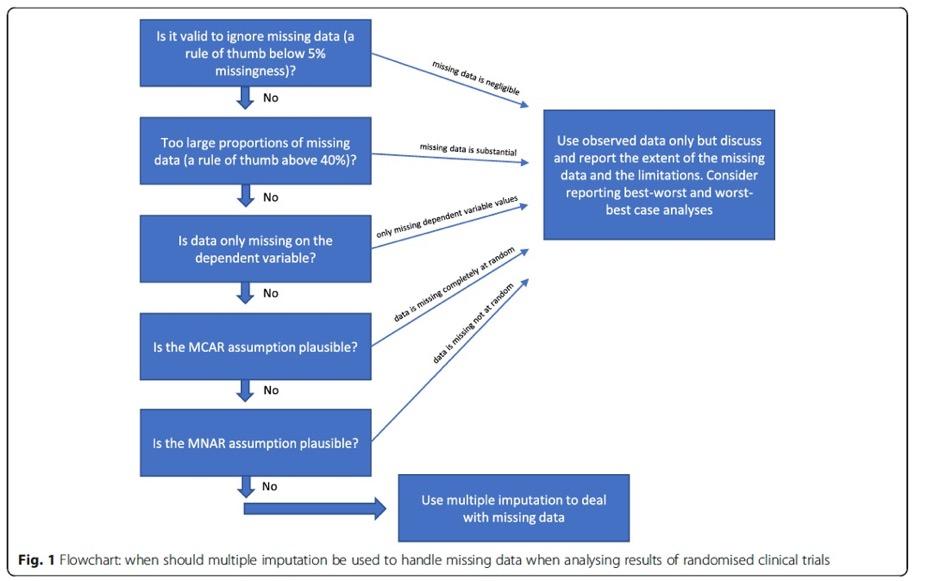

Supplement: Supplementary file 1 — Supplementary Material 1 [file 12876_2024_3247_MOESM1_ESM.docx]
