# Supplementary material for: Serrated polyposis syndrome: defining the epidemiology and predicting the risk of dysplasia
Source: BMC Gastroenterol. 2024 May 16;24:167. doi: 10.1186/s12876-024-03247-2 (PMC11100053; doi:10.1186/s12876-024-03247-2)
Supplement: Supplementary file 3 — Supplementary Material 3 [file 12876_2024_3247_MOESM3_ESM.docx]

Table 1. SPS clinical and endoscopic categorical variables, along with how they will be recorded

| Sex | 1 | Male |
| --- | --- | --- |
|  | 2 | Female |
| WHO diagnostic criteria | 1 | ≥5 serrated lesion proximal to the sigmoid colon with ≥2 polyps ≥10mm |
|  | 2 | Greater than 20 serrated lesions in the colon |
|  | 3 | 1 serrated lesion with a of family history SPS |
| Family history of CRC | 1 | 1^st^ degree |
|  | 2 | 2^nd^ degree |
|  | 3 | No |
|  | 4 | Unknown |
| Patient CRC history | 1 | CRC prior to SPS diagnosis |
|  | 2 | CRC at time of SPS diagnosis |
|  | 3 | CRC post SPS diagnosis |
|  | 4 | No |
| Index colonoscopy | 1 | Yes |
|  | 2 | No |
|  | 3 | Unknown |
| FOBT result | 1 | Positive |
|  | 2 | Negative within 3 months |
|  | 3 | Not performed |
| Smoking status | 1 | Yes |
|  | 2 | No |
|  | 3 | Ex-smoker |
| Diabetes | 1 | Yes |
|  | 2 | No |
| Ethanol consumption | 1 | Yes >30g/d |
|  | 2 | No |
| Other adenomas | 1 | Yes |
|  | 2 | No |
| Types of other adenomas | 1 | TA |
|  | 2 | TVA/VA |
|  | 3 | HP |
|  | 4 | Mixed |
|  | 5 | Other |
|  | 6 | NA |
| Location of CRC | 1 | Right colon |
|  | 2 | Left colon |
|  | 3 | Unknown |
| Location of DSSLs | 1 | Right colon |
|  | 2 | Left colon |
|  | 3 | Unknown |
| Paris morphology | 1 | IIa |
|  | 2 | IIb |
|  | 3 | Is |
|  | 4 | Isp |
|  | 5 | Ip |
| Is component in polyp | 1 | Yes |
|  | 2 | No |
